# Motor Cortex Response to Pleasant Odor Perception and Imagery: The Differential Role of Personality Dimensions and Imagery Ability

**DOI:** 10.3389/fnhum.2022.943469

**Published:** 2022-07-12

**Authors:** Carmenrita Infortuna, Francesca Gualano, David Freedberg, Sapan P. Patel, Asad M. Sheikh, Maria Rosaria Anna Muscatello, Antonio Bruno, Carmela Mento, Eileen Chusid, Zhiyong Han, Florian P. Thomas, Fortunato Battaglia

**Affiliations:** ^1^Department of Biomedical and Dental Sciences and Morphofunctional Imaging, Policlinico Universitario “G. Martino”, University of Messina, Messina, Italy; ^2^Department of Medical Sciences, Hackensack Meridian School of Medicine, Nutley, NJ, United States; ^3^Department of Neurology, Hackensack Meridian School of Medicine, Nutley, NJ, United States; ^4^Department of Art History and Archeology, Italian Academy for Advanced Studies, Columbia University, New York, NY, United States; ^5^Department of Pre-Clinical Sciences, New York College of Podiatric Medicine, New York, NY, United States

**Keywords:** corticospinal excitability, pleasant odor perception, odor imagery, personality traits, transcranial magnetic stimulation (TMS)

## Abstract

**Background:**

Neuroimaging studies have shown a complex pattern of brain activation during perception of a pleasant odor and during its olfactory imagery. To date, little is known regarding changes in motor cortex excitability during these tasks. Bergamot essential oil (BEO) is extensively used in perfumes and cosmetics for its pleasantness. Therefore, to further our understanding of the human sense of smell, this study aimed to investigate the effect of perception and imagery of a pleasant odor (BEO) on motor cortex using Transcranial magnetic stimulation (TMS).

**Materials and Methods:**

We examined the primary motor cortex (M1) excitability during perception of a pleasant odor (BEO) or perception of odorless saline (experiment 1). Furthermore, we tested the effect of olfactory imagery (OI) of BEO on corticospinal excitability (experiment 2). The increase in motor evoked potential (MEP) amplitude was correlated with personality dimensions scores, pleasantness, vividness, and general imagery ability.

**Results:**

The results indicate that the corticospinal excitability changed after both perception and imagery of a pleasant odor (BEO). The correlation analysis shows an association with neuroticism personality trait (experiment 1) and with general olfactory imagery ability (experiment 2).

**Conclusion:**

Both perception of a pleasant odor and its olfactory imagery modulate motor cortex excitability. The enhanced brain activation is affected by specific individual characteristics. Overall, our findings provide physiological evidence for a complex interaction between the olfactory and motor systems.

## Introduction

The olfactory system has ancestral purposes, and it is integrated with other neural structures influencing attention, emotion, memory, and motor control (Riera and Dillin, 2016). For instance, hedonic tagging during olfactory perception guides behaviors such as social interaction, food consumption, reward, and emotional reactivity (Low et al., 2021; Faour et al., 2022; Han et al., 2022). Previous studies investigating brain activation during exposure to pleasant and unpleasant odors reported the presence of a hedonic map with activation of the medial region of the rostral orbitofrontal cortex (Grabenhorst et al., 2007), the posterior part of insula (Wicker et al., 2003), and the anterior cingulate cortex (Callara et al., 2021) after exposure to pleasant odors. Yet, little is known regarding the activation of motor related areas. The investigation of changes of corticospinal excitability during odor perception are of pivotal importance in understanding adaptive motor behaviors during perception of odors (either getting close to or moving away from the odor source according to the hedonic valence). It was demonstrated that some scented essential oils increase cortical excitability and animal studies have demonstrated that they may induce tonic-clonic seizures through the modulation of GABAergic neurotransmission (Bahr et al., 2019). In a study using transcranial magnetic stimulation (TMS), it was suggested that pleasant odors associated with food facilitated the activation of the human motor system (Rossi et al., 2008). Moreover, the interplay between olfactory system and motor system during olfactory hedonic perception were not yet fully investigated. A previous study indicates a functional link between the olfactory bulb and the motor cortex with a specific pattern of oscillations associated with both odor’ valence and avoidance response (Iravani et al., 2021). In addition, the processing of odor pleasantness engages a large network including the precentral gyrus (Ruser et al., 2021). Hence, the use of TMS may provide additional information regarding the functional connectivity between olfactory and motor areas during exposure to a pleasant odor not associated with food. Bergamot Essential Oil (BEO) is derived from *Citrus bergamia*, which is grown primarily in Calabria, Italy. The oil triggers a sensation of pleasantness not strictly associated with food and for this reason is widely used in perfumery, cosmetics, and aromatherapy. Studies indicate that BEO has neuroactive components and may improve mild symptoms of anxiety, depression, and chronic pain (Rombolà et al., 2016; Scuteri et al., 2019). Motor responses to perception of pleasant odor not associated with food might be affected by different factors. For instance, personality traits affect sensory processing and perceptions of one’s environment. Studies indicate that both personality dimensions and cognitive factors modulate odor perception, discrimination, and identification (Larsson et al., 2000; Hedner et al., 2010; Seo et al., 2013). Thus, personality factors need to be considered in studies assessing brain activation and olfactory pleasantness.

Olfactory imagery (OI) is defined as the individual ability to generate the sensations associated with perception without direct olfactory stimulation (Arshamian and Larsson, 2014). Neuroimaging studies indicate that OI engages similar neuronal networks to odor perception (McNorgan, 2012). While neural responses to food odor have been shown to trigger a visual imagery which is an integral part of the “appetizing” aspect of food (Rossi et al., 2008) it remains to be demonstrated whether the OI of a pleasant odor unrelated to food/feeding may activate sensorimotor networks.

Considering the aforementioned research, we sought to ascertain the following: (1) is there an increase in MEP size during pleasant odor perception? (2) Is there an increase in MEP size during olfactory imagery of pleasant odors? and (3) Are these effects associated with specific personality dimensions? We hypothesized that both perception of pleasant odor and olfactory imagery will activate the corticospinal system, and that these effects will be associated with specific personality traits.

## Materials and Methods

### Subjects

Twenty-five right-handed (Oldfield, 1971) healthy subjects (12 women, mean age ± SD: 28.8 ± 7.2) were enrolled in the study. We followed recommended safety guidelines (Rossi et al., 2021) and excluded smokers, those with a history of neuropsychiatric diseases, under treatment with neuroactive drugs, and using alcohol. Furthermore, we excluded subjects who reported an impaired sense of smell due to infection, allergies, sinus pathology, neurodegenerative diseases, and trauma. The study was approved by the Institutional Review Board of the New York College of Podiatric Medicine, New York, NY, United States and conducted in accordance with the Declaration of Helsinki. All subjects completed a written informed consent.

### Experimental Procedure

This was a randomized, placebo-controlled, crossover study. The experimental design consisted of three sessions. During day 1, we assessed whether the participants met the inclusion criteria. The participants enrolled into the study underwent personality assessment (Big 5) and the self-assessment of olfaction Self-reported Mini Olfactory Questionnaire (Self-MOQ). Each subject anonymously completed the 44 items Big Five Inventory (BFI) (John et al., 1991). The personality assessment consisted of 44 short phrases with each response being recorded on a five-point Likert scale (1 = Disagree strongly, 2 = Disagree a little, 3 = Neither agree nor disagree, 4 = Agree a little, 5 = Agree strongly). The surveys were graded by experiments and five psychometrics categories including Extraversion, Agreeableness, Conscientiousness, Neuroticism, and Openness were scored for each subject. The Self-MOQ is a 14 items instrument used for participants’ self-assessment of olfaction. Each of the 14 statements is scored as “totally disagree” score 0, “partially disagree” score 1, “partially agree” score 2, and “totally agree” score 3. Scores below 3.5 indicates a normosmic subject (Rubel et al., 2020; Zou et al., 2020; Fisicaro et al., 2021). We included in the study only participants that scored below this cut-off.

**FIGURE 1:**
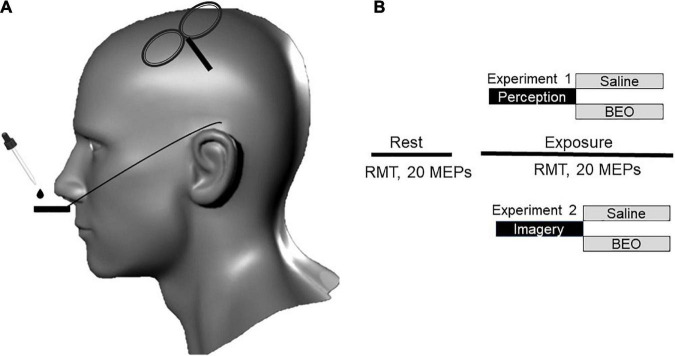
Study design and experimental procedure. **(A)** Transcranial magnetic stimulation (TMS) stimuli were delivered over the left motor cortex. Olfactory stimulation was carried out by using paper strips soddened with Bergamot essential oil (BEO) or with saline. **(B)** In experiment 1 (perception), the participants were asked to inhale two drops of bergamot essential oil of saline positioned on a paper located on metal holder below the nose. The two interventions were randomized and separated by a 2-h break. During experiment 2 (Odor imagery), the participant was asked to mentally rehearse (odor imagery) the two conditions experienced the in the previous day. At rest, we recorded resting motor threshold (RMT) and twenty motor evoked potentials (MEPs). The same parameters were tested during the continuous exposure/imagery of BEO/saline.

During day 2 (“perception”), we investigated the effect of bergamot essential oil (BEO) perception on Resting Motor Threshold (rMT) and Motor Evoked Potentials (MEP) amplitude. Subjects in the experimental group were required to nasally inhale the scent emitted from prepared fragrance blotter strips with BEO. To prepare these scented strips, two drops (0.1 mL) of BEO were placed on sterile fragrance blotter strips positioned on a metal holder (placed 1 cm below the nose). In the control condition we used the same amount of saline solution (Figure 1). According to the manufacturer (Essenze Bova 1997, Melito di Porto Salvo, Reggio Calabria, Italy), the oil was extracted via cold-pressed methods from Bergamot (Citrus bergamia) from Italy’s Calabria region. The active molecules in the BEO were characterized in a previous study (Sicari et al., 2016). The dose of BEO used in this study was in keeping with dosages used in clinical trials (Peng et al., 2009). After the experiment the participant were asked to rate pleasantness on a Visual Analog Scale (VAS) scale (from 0 = “extremely unpleasant” to 100 = “extremely pleasant”).

On day 3 (“olfactory imagery), we first assessed the vividness of olfactory imagery using the vividness of olfactory imagery questionnaire (VOIQ) (Gilbert et al., 1988). Briefly, the VOIQ is a 16-items instrument split into four categories of odors: personal hygiene, food-related, tobacco, and vehicles. For each category, the participants were asked to image 4 different related odors and rate them on a Likert scale: (1) perfectly realistic and as vivid as the actual odor; (2) realistic and reasonably vivid; (3) moderately realistic and vivid; (4) vague and dim; (5) no odor at all, you only “know” that you are thinking of the odor. The mean VOIQ score was calculated for each participant (sum of answers/16). The vividness of BEO imagery was tested by using the same Likert scale. We then assessed the effect of BEO OI and Saline OI on rMT and MEP amplitude using the same experimental design.

### Electromyographic Recording

We recorded motor evoked potentials (MEPs) using surface electrodes positioned in a tendon-belly arrangement on the right abductor pollicis brevis (APB) muscle at rest. Surface EMG was monitored simultaneously to ensure muscle relaxation. The signal was amplified, filtered (band-pass 2 Hz to 5 kHz), digitized at 5 kHz (Micro1401, Cambridge Electronics Design, Cambridge, United Kingdom), and collected for analysis.

### Cortical Excitability

Participants were seated comfortably in an armchair with a comfortable arm support. TMS was delivered using a Magstim 200 stimulator and a figure-of-eight coil (9 cm) (The Magstim Company, Dyfed, United Kingdom). The coil was optimally positioned over the primary motor cortex at a 45° angle away from the midline. The rMT was determined according to the recommendations of the International Federation of Clinical Neurophysiology (Rossini et al., 2015) and defined as the minimum stimulation intensity capable of generating resting MEP of at least 50 μV in amplitude in 5 of 10 trials. Upon determination of each subject’s rMT, twenty MEPs were collected, measured from peak-to-peak, and averaged using a stimulation intensity that was 120% of the rMT (Cantone et al., 2019). Each subject was tested in the morning (9 AM–10 PM) in two different consecutive days. During day 2, we assessed changes in rMT and MEP amplitude at rest and during olfactory stimulation with either saline or BEO. During day 3, we tested the same electrophysiological parameter before and after OI of the same conditions (saline/BEO). The control and experimental conditions were randomized. RMT and MEP amplitude were first recorded at rest (without exposure to BEO/Saline) and then during the exposure or imagery to BEO/saline.

### Statistical Analysis

The data distribution was assessed using the Kolmogorov–Smirnov test. *T*-test was used to compare means at rest. Differences in rMT and MEP amplitudes were assessed using a repeated-measure ANOVA (main effect: Condition and Time). When variables were not normally distributed values log(*x* + 1) transformed for the repeated-measure ANOVA. If the assumption of sphericity was violated in the Mauchly’s sphericity test, the Greenhouse–Geisser correction was used. *Post hoc* comparisons using paired-samples *t*-test were used to evaluate the effects of perception and olfactory imagery on MEP amplitude. Spearman’ Rho’ correlation coefficient was used to assess the correlation between variables. We used SPSS version 23 Software (SPSS Inc., Chicago, IL, United States) for statistical analysis and *p*-values were considered statistically significant when <0.05.

## Results

The experiments were well-tolerated without any adverse effect. Participants’ pleasantness of BEO score, VOIQ score, vividness of BEO OI, and personality dimensions’ scores are reported in Table 1.

**TABLE 1:** Mean ± SD scores for pleasantness of bergamot essential oil (BEO) perception, vividness of olfactory imagery questionnaire (VOIQ), vividness of BEO olfactory imagery (OI), and Big 5 personality dimensions.

	Pleasantness of BEO	VOIQ score	Vividness of BEO OI	Extraversion	Agreeableness	Conscientiousness	Neuroticism	Openness
Mean	76.36	32.72	1.92	3.24	3.53	3.02	3.47	3.71
SD	14.5	8.4	0.86	0.78	0.87	1.21	103	0.66

**FIGURE 2:**
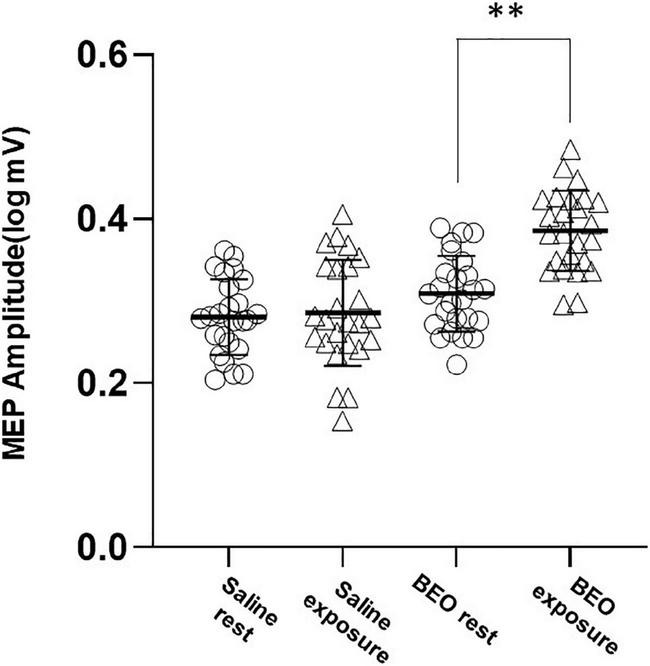
Experiment 1 (perception): average amplitudes of logMEP before and after perception of either Saline or BEO. Perception of BEO significantly increased the amplitude of logMEP. Data are mean ± SD. **p < 0.01.

Motor evoked potential values were not normally distributed and were log transformed for the analysis. We first tested the effect of perception of a pleasant odor on rMT and MEP amplitude (Experiment 1). At baseline, both rMT and MEP amplitude were not different in the two conditions (Saline: rMT = 40.28 ± 6.48%, BEO rMT: 40.42 ± 6.1%, *p* = 0.03; Saline logMEP: 0.28 ± 0.08 V, BEO logMEP: 0.31 ± 0.09 V, *p* = 0.14). Exposure to BEO and saline did not change rMT [main effect *Time:* Wilks’ lambda: 0.97, *F*_(1_,_48)_ = 1.39, *p* = 0.24, η^2^ = 0.02; *Group*× *Time* interaction: Wilks’ lambda: 0.97, *F*_(1_,_48)_ = 1.04, *p* = 0.31, η^2^ = 0.02]. Regarding the MEP amplitude, the two-way RMANOVA results indicate a statistically significant main effect of *Time*, sphericity assumed [Wilks’ lambda: 0.57, *F*_(1_,_48)_ = 35.99, *p* < 0.001, η^2^ = 0.429] and a significant *Group*× *Time* interaction, sphericity assumed [Wilks’ lambda: 0.63, *F*_(1_,_48)_ = 327.57, *p* < 0.001, η^2^ = 0.365]. *Post hoc* analysis indicated that MEPs were significantly larger during perception of BEO compared to saline (*t* = −7.14, *p* < 0.0001) (Figure 2).

**FIGURE 3:**
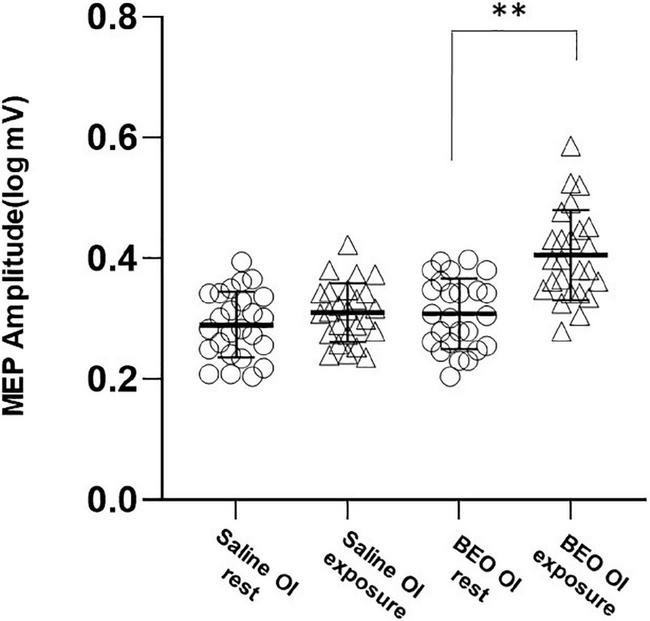
Experiment 2 (odor imagery): average amplitudes of logMEP before and after imagery of either Saline or BEO. Imagery of BEO significantly increased the amplitude of logMEP. Data are mean ± SD. **p < 0.01.

We next examined corticospinal activation during BEO OI (experiment 2). No changes in rMT were detected [main effect *Time:* Wilks’ lambda: 0.9, *F*_(1_,_48)_ = 2.33, *p* = 0.71 η^2^ = 0.03; *Group*× *Time* interaction: Wilks’ lambda: 0.99, *F*_(1_,_48)_ = 0.13, *p* = 0.71, η^2^ = 0.03]. On the contrary, olfactory imagery of BEO increased MEP size [main effect of *Time*, sphericity assumed, Wilks’ lambda: 0.52, *F*_(1_,_48)_ = 43.1, *p* < 0.001, η^2^ = 0.47; and a significant *Group*× *Time* interaction, sphericity assumed: Wilks’ lambda: 0.72, *F*_(1_,_48)_ = 18.18, *p* < 0.001, η^2^ = 0.315]. *Post hoc* analysis indicated that MEPs were significantly larger during imagery of BEO compared to saline (*t* = −5.24, *p* < 0.0001) (Figure 3). RMT and MEP amplitude were not different at baseline (Saline OI: rMT = 39.8 ± 7%, BEO OI: rMT: 40 ± 6.2%, *p* = 0.43; Saline logMEP: 0.29 ± 0.1, BEO logMEP: 0.31 ± 0.1, *p* = 0.17).

**FIGURE 4:**
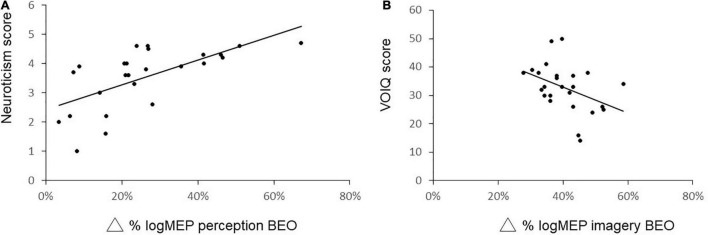
Correlation analysis between changes in logMEP amplitude after perception of BEO and Neuroticism score **(A)** and changes in logMEP amplitude after odor imagery and vividness of olfactory imagery questionnaire (VOIQ) score **(B)**. Lower VOIQ scores indicate better imagery ability.

We next correlated the increase in logMEP size after perception of BEO (Δ BEO perception: 26 ± 16%) with pleasantness of BEO (*r* = −0.07, *p* = 0.72), extraversion score (*r* = −0.04, *p* = 0.824), agreeableness score (*r* = −0.016, *p* = 0.44) conscientiousness score (*r* = 0.1, *p* = 0.1), neuroticism score (*p* = 0.73, *p* < 0.01), openness score (*r* = 011, *p* = 0.58). Also the increase in logMEP amplitude after BEO OI (Δ BEO imagery: 36 ± 25%) was correlated with the VOIQ score (*r* = −0.47, *p* = 0.02), vividness of BEO OI (*r* = 0.01, *p* = 0.95), extraversion score (*r* = −0.31, *p* = 0.12), agreeableness score (*r* = 0.09, *p* = 0.64), conscientiousness score (*r* = 0.07, *p* = 0.71), neuroticism score (*r* = −0.27, *p* = 0.18), openness scores (*r* = −0.21, *p* = 0.3). Thus, the correlation analysis indicates that the neuroticism traits are associated with larger MEPs after BEO perception (Figure 4A) while the facilitatory effect of BEO OI on motor cortex is linked to the individual general olfactory imagery ability (Figure 4B).

## Discussion

In this study, we analyzed relationships amongst olfactory perception, olfactory imagery, personality dimensions, and motor system. We found that both perception of a pleasant odor (BEO) and its mental rehearsal increase MEP amplitude. Furthermore, the increase in corticospinal excitability is correlated with specific personality dimensions and with imagery ability.

The increase of MEP after exposure to a pleasant odor we observed is in keeping with a previous report (Rossi et al., 2008) showing similar findings after sniffing alimentary odorants indicating that pleasant smells (both food and non-food related) activate the motor system. Exposure to food odors can trigger physiological (visual imagery and grasping) (Rossi et al., 2008), and biochemical (hormones, enzymes, and neurotransmitters) adaptations (Smeets et al., 2010) in anticipation of food consumption. By contrast, since the participants did not associate BEO to food, we can assume that the reported cortical activation after sensory stimulation depends upon the hedonic nature of the odor. There are several possible mechanisms underlying our results. It was demonstrated that systemic administration of BEO elevates the concentration of extracellular levels of aspartate, glycine, and taurine in the hippocampus of freely moving rats (Morrone et al., 2007). Furthermore, this effect is Ca2+-dependent suggesting that BEO may increase the presynaptic release of the excitatory amino acids (Morrone et al., 2007). BEO can induce behavioral arousal in rats (increased sniffing, grooming, and exploratory activity) which is associated with a cortical increase of power density in the alpha and beta frequency (Rombolà et al., 2009). Although these preclinical results were obtained using intraperitoneal BEO injections, evidence indicates that BEO administered via inhalation is pharmacologically active on the rodent brain (Scuteri et al., 2022) and that volatile components of BEO can reach the limbic system directly through the olfactory system or passing through the alveoli into the capillaries in humans (Brooker et al., 1997). Thus, it is conceivable that the effect on MEP amplitude may be due to the modulation of excitatory and inhibitory circuits in M1. In addition, it was demonstrated that occurrence of EEG beta rhythm before a magnetic stimulus is associated with the manifestation of MEP of a larger amplitude (Hussain et al., 2019). These data support the hypothesis that the increase in mean beta power induced by BEO could inhibit M1 layer II/III interneurons and excite layer V corticospinal neurons inducing, in this way, corticospinal gain output (Khademi et al., 2018). Future studies using different TMS and EEG paradigms are needed to further investigate this research hypothesis. A recent meta-analysis investigating the brain activation during perception of pleasant odor across studies indicated consistent activation of bilateral amygdala, orbitofrontal cortex (OFC), piriform cortex, insula, pallidum, putamen, and the central operculum (Torske et al., 2022). According to this activation pattern, it is likely that the hedonic encoding activates both the reward system (Okuda et al., 2003) and the corticostriatal loops subserving the stimulus-action-dependent reward circuits (Haruno and Kawato, 2006; Smith et al., 2009) which may explain the corticospinal activation to pleasantness we recorded. We can also speculate that the pleasantness of the sensory experience might convey a sense of beauty and consequent aesthetic processing which has been previously associated with limbic system activation, increases in motor cortex excitability, changes in connectivity, and sensorimotor integration (Battaglia et al., 2011; Concerto et al., 2015, 2016; Mineo et al., 2018b) indicating that motor cortex integrates information about rewards and prepares to performance (Galaro et al., 2019; Di Lazzaro et al., 2021).

We found that OI of BEO had a facilitatory effect on the corticospinal system. During mental imagery we simulate perceptual experiences. Previous works indicate that OI (like olfactory perception) activates primary olfactory cortex alongside limbic structures such as hippocampus, anterior cingulate cortex, and insula (McNorgan, 2012). Thus, the increases in MEP amplitude after BEO OI might rely upon the modulation of the same networks we have previously discussed explaining MEP gain during BEO perception. Studies have consistently revealed that the human imagery system comprises both modality-specific and modality-independent components (Daselaar et al., 2010). Regarding imagery of pleasant odors, it has been hypothesized that the olfactomotor activity during OI mimics that during perception (Bensafi et al., 2003) and that the process by which an olfactory image is created might depend upon the induction of nasal inhalation. Thus, the greater airflow during olfactory imagery (sniffing) became an essential component of the cortical activation necessary for odor encoding (Bensafi et al., 2003) and indicates activation of motor areas.

Although correlation does not mean causal link, our results point to a role of neuroticism traits and general olfactory imagery capability as important factors associated with motor cortex activation during the tasks. Nonetheless, it is to be explained why neuroticism traits during perception of BEO would induce stronger motor cortex activation. Given that research has shown that chemosensory sensitivity is linked to higher neuroticism traits scores (Kärnekull et al., 2011), it is possible that the higher emotional and cognitive reactivity associated with this trait might underlie this effect. For instance, during odor perception participants with high neuroticism trait display stronger activation in cingulate cortex (Hillert et al., 2007), a brain area thought to play a pivotal role in the interplay between emotion, cognition, and motor control (Paus, 2001). Hence, activation of limbic structure during perception and imagery of a peasant odor might trigger emotional arousal and consequently increase MEP amplitude (Borgomaneri et al., 2021). The strong connections between the olfactory nerve, the amygdala, and the hippocampus (Herz, 2016) support the evidence indicating that odor triggers autobiographical memories (El Haj, 2022), which have been associated with increased motor cortex activation (Mineo et al., 2018a). Thus, we can hypothesize a contribution of memory networks to the activation of the motor cortex during BEO perception and imagery. Regarding OI, the correlation of the MEP gain with imagery abilities we observed is consistent with the literature. Neuroimaging studies indicate that the participant’s difference in OI ability is mirrored in a different pattern of brain activation (Plailly et al., 2012). Future studies should address the role of expertise, odor expectation, and rehearsal of information previously associated with odor in OI facilitation of motor areas (Royet et al., 2013).

Furthermore, we cannot entirely exclude that some participants might associate BEO to food. Thus, the gut-brain interactions and their contribution to hyperexcitability (Pennisi et al., 2017; Lanza et al., 2018) should be further investigated to determine cortical responses to olfactory stimulation. Furthermore, the study of neural responses to odors perception and imagery with non-invasive brain stimulation techniques may provide useful information to better understand olfactory dysfunction and olfactory imagery deficits associated with COVID-19 infections (Tomasino et al., 2022).

### Limitations

The current study has limitations. First, the study design did not allow us to perform blind experiments. Future studies using different experimental conditions (dose-response, different essential oils, and different TMS paradigms) are needed to further explore the effects of olfactory perception and imagery on motor cortex excitability. In addition, there are important variables that need to be considered to avoid biases. For instance, all our participants were college students. It was demonstrated that academic stress is highly prevalent in students (Infortuna et al., 2020a) and affects motor cortex plasticity (Concerto et al., 2017b,^2018^) and metaplasticity (Infortuna et al., 2021). Furthermore, health-risk behaviors in college students may affect cortical excitability (Concerto et al., 2017a; Mineo et al., 2018c; Infortuna et al., 2020b), and sleep quality (Sawah et al., 2015). We believe that the cross-over design of our study minimized possible biases; nonetheless, future studies should consider these factors.

An important aspect that was not investigated in our study is the contribution of subcortical sites (brainstem and spinal cord) to corticospinal facilitation. We used a rather strong olfactory stimulation that might have induced sniffing and consequent teeth clenching. A previous study indicates that teeth clenching facilitates corticospinal excitability at different levels: cortical, brainstem, and spinal (Boroojerdi et al., 2000). Thus, future studies should consider temporalis muscle and masseter muscle EMG monitoring to exclude trials with teeth clenching and include brainstem and spinal excitability protocols to ascertain the subcortical contribution to MEP facilitation. The use of paired-pulse paradigms could further our understanding regarding the role of intracortical excitatory and inhibitory circuits in facilitating MEPs during olfactory imagery and perception.

## Conclusion

The study was well-powered to detect medium effect sizes. Our results provide novel evidence for a modulatory effect of pleasant odor perception and imagery on motor networks. Furthermore, our data point toward a pivotal role of personality traits and individual imagery abilities in mediating such effects. Future studies should address the clinical relevance of our results.

## Data Availability Statement

The raw data supporting the conclusions of this article will be made available by the authors, without undue reservation.

## Ethics Statement

The studies involving human participants were reviewed and approved by the Institutional Review Board of the New York College of Podiatric Medicine, New York, NY, United States. The patients/participants provided their written informed consent to participate in this study.

## Author Contributions

CI, FG, SP, AS, and FB contributed to the data acquisition and analysis. DF, MM, AB, CM, EC, ZH, FT, and FB contributed to the interpretation of the data. CI and FB designed the experiments and drafted the manuscript. All authors contributed to the article, and reviewed and approved the submitted version.

## Conflict of Interest

The authors declare that the research was conducted in the absence of any commercial or financial relationships that could be construed as a potential conflict of interest.

## Publisher’s Note

All claims expressed in this article are solely those of the authors and do not necessarily represent those of their affiliated organizations, or those of the publisher, the editors and the reviewers. Any product that may be evaluated in this article, or claim that may be made by its manufacturer, is not guaranteed or endorsed by the publisher.
